# Gender and Institutional Differences in Preferred Resident Responses to Biased Statements in Medical Education: A Descriptive, Cross‐Sectional Analysis

**DOI:** 10.1002/aet2.70206

**Published:** 2026-06-09

**Authors:** Jeff Druck, Emad Awad, Dustin Blake Williams, William Harousseau

**Affiliations:** ^1^ Department of Emergency Medicine University of Utah Spencer Fox Eccles School of Medicine Salt Lake City Utah USA; ^2^ Department of Emergency Medicine University of Texas Southwestern Medical Center Dallas Texas USA

## Abstract

**Background:**

Bias and microaggressions remain persistent challenges in medical education, threatening psychological safety and perpetuating inequities. How learners respond to these incidents and the influence of gender and institutional culture on these responses remain underexplored.

**Objective:**

To evaluate preferred strategies for responding to biased statements among emergency medicine residents, comparing differences by gender and institutional affiliation.

**Methods:**

In this cross‐sectional study, an anonymous survey was distributed to residents at two academic institutions: the University of Utah and UT Southwestern. Participants ranked five possible responses to biased comments originating from patients, staff, peers, and learners. Descriptive statistics and Mann–Whitney *U* tests were used to analyze differences by gender and institution.

**Results:**

Among 100 respondents (52% female), the majority preferred active engagement—either addressing the bias directly or collaborating with an attending—across all scenarios. Ignoring bias was consistently the least preferred option. Gender differences were significant in responses to biased staff statements: men showed a stronger preference for addressing the issue themselves (*p* = 0.005), while women showed a stronger preference for collaborating with an attending (*p* = 0.03). Institutional differences were also observed (*p* < 0.05). UT Southwestern residents more often favoring attending‐led/collaborative approaches in patient and staff scenarios, whereas University of Utah residents more often favored addressing bias themselves.

**Conclusions:**

Residents generally favored proactive responses to biased statements, with distinct differences based on gender and institutional culture. These findings suggest the need for tailored bias response training that considers both individual and organizational factors. Further research should explore how other identity variables (e.g., race, sexual orientation) intersect with response preferences and how institutions can foster environments that empower all learners to address bias confidently.

## 1 Background

Bias and microaggressions in medical education continue to present substantial challenges to creating inclusive and equitable learning environments [1]. These experiences—whether involving race, gender, or other aspects of identity—can undermine psychological safety, affect professional identity formation, and perpetuate structural inequities within the medical profession [2]. Educators and institutions play a pivotal role in setting the tone for how such behaviors are addressed. However, understanding learners' preferred responses to biased statements is equally important, particularly as they transition from medical school to residency and take on more prominent roles in clinical teams.

Existing literature has shown that a complex interplay of personal characteristics and institutional culture influences responses to microaggressions. A qualitative study by Haykal et al. [3] highlighted that both faculty members and trainees experienced biased behaviors in academic medicine, emphasizing the importance of institutional support and structured policies in shaping responses. Similarly, Wittels et al. [4] found evidence of implicit gender and racial biases among academic emergency medicine faculty, underscoring the importance of considering identity‐based factors such as gender when exploring how individuals respond to discrimination.

In addition, perceptions of how institutions handle bias can shape whether learners feel empowered to speak up. Williams et al. [5] demonstrated that, even within the same department, faculty and trainees differed in their experiences with discrimination and in their trust in institutional responses. These findings suggest that institutional context—not just individual attitudes—may significantly affect the strategies learners prefer when encountering biased remarks.

Despite these insights, there is limited empirical research that directly compares learners at different stages of training or across various institutions. Similarly, few studies have explored how gender identity may influence preferred strategies for handling bias. Understanding these differences is essential for tailoring bias response training to meet learners' specific needs and contexts.

To address these gaps, this study aimed to: (1) evaluate differences in preferred strategies for addressing biased statements between residents based on location, (2) assess gender‐based differences in approaches to managing bias, and (3) compare responses between learners at two institutions—University of Utah and UT Southwestern—with potentially distinct institutional climates and support structures.

## 2 Methods

### 2.1 Study Design and Participants

This cross‐sectional study aimed to explore the perspectives and experiences of residents across diverse training institutions. Participants who met the following criteria were recruited: residents in Emergency Medicine at UT Southwestern and the University of Utah during the 2024–25 academic year. Recruitment was conducted via personal outreach, email communication, and QR codes, with all Emergency Medicine residents from both programs identified as eligible participants. Both the University of Utah and UT Southwestern are known for excellent training in emergency medicine, with both located in urban centers, but with dramatically different sizes, with the University of Utah seeing 80,000 patients a year and the UT Southwestern collaborative seeing 240,000. The residency sizes of the programs vary; both are 3‐year programs, but the University of Utah takes 12 residents a year while UT Southwestern accepts 26 residents a year. As expected, comparing a southern‐based program with a western program reveals differences in attitudes and expectations associated with the respective locales.

### 2.2 Survey Administration

The survey was developed and administered electronically via the Qualtrics platform, a secure and widely used tool for online data collection (see Appendix A for the survey instrument). The survey was designed to capture demographic data and training experiences, focusing on elements such as gender, sexual orientation, race, geographic location, and choice of bias‐focused strategies. Questions included multiple‐choice, Likert‐scale, and open‐ended formats to gather comprehensive data. The survey underwent face validity testing and pilot testing via six faculty members, with two faculty members experienced in survey methodology evaluating the survey and providing feedback. The general categories of biased statements were responses of residents on how they wanted biased statements against them to be addressed by faculty. Table 1 shows the possible options for each scenario:

**TABLE 1:** Initial scenario, observed by group, possible options for addressing the issue.

Biased statement by	Observed by	Method of addressing the statement
Patient	Faculty	Collaboration with faculty
Staff		Have the attending address you
Peer		Have the attending address the patient
Year lower than you		Address it yourself
		Ignore it

### 2.3 Incentives and Recruitment Strategies

Co‐investigators (Co‐Is) at each participating site oversaw recruitment and facilitated incentives tailored to their respective institutional resources. In Utah, each resident who completed the survey, verified only by the honor system, received a $5 Starbucks gift card. At UT Southwestern, an additional 200 dollars was added to the end of year party fund if there was a greater than 80% response rate. To maximize participation, site‐specific Co‐Is sent reminder emails to the resident groups. All surveys were sent from December 2024 to January 2025, during the academic year 2024–25.

### 2.4 Data Collection and Handling

All data were collected anonymously, with no identifying information linked to responses. Each institution received a de‐identified dataset containing responses specific to their site.

### 2.5 Ethical Considerations

This study was submitted to the University of Utah IRB office, where it was determined to meet the IRB exemption criteria (IRB number 00182023) before any data collection took place. All participants provided informed consent before participating in the survey by continuing to participate. The anonymity of participants was maintained throughout data collection, analysis, and dissemination of results.

## 3 Statistical Analysis

### 3.1 Data Analysis

Descriptive statistics were calculated to summarize participant demographics, including gender, race, and institutional affiliation (University of Utah vs. UT Southwestern). Categorical variables were reported as percentages. For each scenario (patient, staff, peer, learner), participants ranked five response strategies from 1 (most preferred) to 5 (least preferred). We report rank‐frequency distributions and medians (IQR). To provide an overall ordering of preferences within each scenario, we calculated a weighted average preference score (Rank 1 = 5 through Rank 5 = 1), with higher scores indicating greater overall preference. Group differences by gender and institution were assessed using the Mann–Whitney U test for ordinal data; direction was interpreted using medians, where a lower median rank indicates a stronger preference. Statistical significance was set at *p* < 0.05. All analyses were performed using SPSS version 30 (IBM, Armonk, NY).

## 4 Results

### 4.1 Descriptive Statistics

The total study population was 114 possible respondents (36 University of Utah Emergency Medicine Residents and 78 Emergency Medicine residents at UT Southwestern). Initially, we had 105 participants; however, five were excluded because they had missing data on a key variable (race) required for analysis. The remaining 100 participants (*N* = 100) were included in the study and had complete responses. Of those, 52% were females and 48% were males. The racial composition was predominantly White (70%), followed by Asian (16%), Hispanic or Latino (10%), and Black or African American (4%). In terms of institutional representation, 71% of participants were from UT Southwestern, while 29% were from the University of Utah (Table 2). Overall, this represents a response rate of 87.7% (100/114), which was divided as 71/78 residents at UT Southwestern (91% response rate) and 29/36 residents at the University of Utah (80.6% response rate).

**TABLE 2:** Study participants.

	Frequency
Gender	
Female	52%
Male	48%
Race	
Asian	16%
Black or African American	4%
Hispanic or Latino	10%
White	70%
Location	
University of Utah	29%
UT Southwestern	71%

Overall preference patterns are summarized using weighted average scores in Table 3, with detailed rank‐frequency distributions shown in Table 4.

**TABLE 3:** Overall preference for response strategies to biased statements, by source (weighted average).

Source of bias	Most preferred (Wt. Avg)	2nd	3rd	4th	Least preferred (Wt. Avg)
Patient	Collaborate with attending (3.85)	Address it yourself (3.53)	Have attending address patient (3.23)	Attending says to you only (2.44)	Ignore it (1.90)
Staff	Collaborate with attending (3.81)	Address it yourself (3.70)	Have attending address staff (3.41)	Attending says to you only (2.40)	Ignore it (1.72)
Peer	Address it yourself (4.12)	Collaborate with attending (3.69)	Have attending address peer (3.07)	Attending says to you only (2.51)	Ignore it (1.71)
Learner	Address it yourself (4.34)	Collaborate with attending (3.65)	Have attending address learner (3.06)	Attending says to you only (2.49)	Ignore it (1.50)

**TABLE 4:** Frequency distributions and weighted average scores of response preferences, by source of bias.

Response strategy	Rank 1	Rank 2	Rank 3	Rank 4	Rank 5	Median (IQR)	Wt. Avg.
Biased patient (*N* = 100)							
Have attending address patient	21	**27**	19	20	13	3 (2–4)	3.23
Address it yourself	26	**29**	23	16	6	2 (1–3)	3.53
Ignore it	12	6	6	12	**64**	5 (4–5)	1.90
Collaborate with attending	**38**	28	18	13	3	2 (1–3)	3.85
Attending says to you only	2	9	33	**43**	13	4 (3–4)	2.44
Biased staff (*N* = 99)							
Have attending address staff	**28**	18	**28**	17	8	3 (1–3.5)	3.41
Address it yourself	**41**	17	17	18	6	2 (1–3)	3.70
Ignore it	5	8	8	11	**67**	5 (4–5)	1.72
Collaborate with attending	22	**47**	21	7	2	2 (2–3)	3.81
Attending says to you only	4	9	25	**46**	15	4 (3–4)	2.40
Biased peer (*N* = 100)							
Have attending address peer	14	**28**	23	21	14	3 (2–4)	3.07
Address it yourself	**61**	12	9	14	4	1 (1–3)	4.12
Ignore it	8	5	5	14	**68**	5 (4–5)	1.71
Collaborate with attending	18	**46**	25	9	2	2 (2–3)	3.69
Attending says to you only	3	8	37	**41**	11	4 (3–4)	2.51
Biased learner (*N* = 100)							
Have attending address learner	12	**30**	22	24	12	3 (2–4)	3.06
Address it yourself	**70**	10	5	14	1	1 (1–2)	4.34
Ignore it	7	2	5	6	**80**	5 (5–5)	1.50
Collaborate with attending	13	**48**	30	9	0	2 (2–3)	3.65
Attending says to you only	0	9	37	**48**	6	4 (3–4)	2.49

*Note:* The bolded values are the highest answer number for each response strategy.

Biased statements from patients: Using weighted average preference scores to summarize overall rank order, residents most preferred collaborating with the attending (Wt. Avg 3.85), followed by addressing it yourself (3.53) and having the attending address the patient (3.23). Ignoring the statement was least preferred (1.90; median 5 [IQR 4–5]) (Tables 3 and 4).

Biased statements from staff members: The preferred responses were addressing the issue themselves (rank 1 = 41%) and collaborating with the attending (rank 2 = 47%). The median ranking (IQR) was 2 (1–3). Ignoring the statement remained the least desired option (rank 5 = 67%; median = 5, IQR = 4–5).

Biased statements from peers: In cases of biased statements from a peer, participants most frequently preferred to address it themselves (rank 1 = 61%), with attending collaboration as the next favored option (rank 2 = 46%). The median ranking (IQR) for these responses was 1 (1–3) and 2 (2, 3), respectively. Ignoring the bias was again the least preferred option (rank 5 = 68% median = 5, IQR = 4–5) (Table 3).

Biased statements from learners: Regarding biased statements from a learner, independent intervention was most preferred (rank 1 = 70%), followed by attending collaboration (rank 2 = 48%), with a median ranking of 1 (IQR = 1–2) and 2 (IQR = 2–3), respectively. Ignoring the statement remained the least favored approach (rank 5 = 80%) (median = 5, IQR = 5–5) (Table 3).

Overall, the results suggest that Emergency medicine residents consistently preferred active engagement, either by addressing the bias themselves or involving the attending rather than ignoring such statements. This preference (against ignoring) was particularly pronounced when bias originated from colleagues or learners.

### 4.2 Gender Differences in Responses to Biased Statements

The Mann–Whitney *U* test was conducted to assess gender differences in responses to biased statements observed by faculty members in various contexts, including those about patients, staff, peers, and learners. Gender differences were not observed for biased patient, peer, or learner scenarios (all *p* > 0.05). For biased staff statements, significant gender differences were observed for two strategies: men showed a stronger preference for addressing the bias themselves (male median 1 [IQR 1–3] vs. female median 3 [IQR 1–4], *p* = 0.005), while women showed a stronger preference for collaborating with an attending (female median 2 [IQR 1–2.2] vs. male median 2 [IQR 2–3], *p* = 0.034) (Table 5).

**TABLE 5:** Gender differences in preferred responses to biased statements (Mann–Whitney *U* test).

Response strategy	Female median (IQR)	Male median (IQR)	*p*
Biased patient			
Have attending address patient	3 (1.8–4)	2.5 (2–4)	0.745
Address it yourself	2 (2–4)	2 (1–3)	0.227
Ignore it	5 (4–5)	5 (3.8–5)	0.391
Collaborate with attending	2 (1–3)	2 (1–3)	0.729
Attending says to you only	4 (3–4)	4 (3–4)	0.641
Biased staff			
Have attending address staff	2.5 (1–3)	3 (1–4)	0.644
Address it yourself	3 (1–4)	1 (1–3)	0.005
Ignore it	5 (4–5)	5 (4–5)	0.676
Collaborate with attending	2 (1–2.2)	2 (2–3)	0.034
Attending says to you only	4 (3–4)	4 (3–4)	0.352
Biased peer			
Have attending address peer	3 (2–4)	3 (2–4)	0.952
Address it yourself	1 (1–3)	1 (1–2.2)	0.254
Ignore it	5 (4–5)	5 (4–5)	0.302
Collaborate with attending	2 (2–3)	2 (2–3)	0.589
Attending says to you only	3 (3–4)	4 (3–4)	0.599
Biased learner			
Have attending address learner	3 (2–4)	3 (2–4)	0.986
Address it yourself	1 (1–2)	1 (1–2)	0.576
Ignore it	5 (5–5)	5 (5–5)	0.353
Collaborate with attending	2 (2–3)	2 (2–3)	0.372
Attending says to you only	4 (3–4)	3.5 (3–4)	0.380

*Note:* Lower median rank indicates stronger preference. Staff scenario uses available cases (*N* = 99; one male respondent missing staff rankings).

### 4.3 Institutional Differences in Preferred Responses to Biased Statements: University of Utah Versus UT Southwestern

Institutional differences were observed across multiple scenarios (Table 6). For biased patient statements, UT Southwestern residents showed a stronger preference for attending‐led/collaborative approaches (e.g., having the attending address the patient and collaborating with an attending), whereas University of Utah residents more often preferred addressing the bias themselves and showed more variable rankings for ignoring the statement, although it remained among the least preferred options. For biased staff statements, UT Southwestern residents more strongly preferred having the attending address the staff member, while Utah residents more strongly preferred addressing the issue themselves. Differences were also observed for learner‐related scenarios, where UT Southwestern residents more strongly preferred collaborating with an attending, whereas Utah residents more strongly preferred addressing the issue themselves (Table 6).

**TABLE 6:** Institutional differences in preferred responses (University of Utah vs. UT Southwestern; Mann–Whitney *U* test).

Response strategy	Univ. of Utah Median (IQR)	UT Southwestern Median (IQR)	*p*
Biased patient (Utah *n* = 29, UTSW *n* = 71)			
Have attending address patient	4 (2–5)	2 (2–3.5)	0.008
Address it yourself	2 (1–3)	3 (2–4)	0.014
Ignore it	4 (2–5)	5 (4–5)	0.001
Collaborate with attending	3 (2–4)	2 (1–2.5)	< 0.001
Attending says to you only	4 (3–4)	4 (3–4)	0.291
Biased staff (Utah *n* = 29, UTSW *n* = 70)			
Have attending address staff	3 (2–4)	2 (1–3)	0.007
Address it yourself	1 (1–2)	2 (1–4)	0.003
Ignore it	5 (3–5)	5 (4–5)	0.132
Collaborate with attending	2 (2–3)	2 (2–2.75)	0.15
Attending says to you only	4 (4–4)	4 (3–4)	0.172
Biased peer (Utah *n* = 29, UTSW *n* = 71)			
Have attending address peer	3 (2–4)	3 (2–4)	0.515
Address it yourself	1 (1–1)	1 (1–3)	0.007
Ignore it	5 (4–5)	5 (4–5)	0.898
Collaborate with attending	3 (2–3)	2 (2–3)	0.088
Attending says to you only	4 (3–4)	3 (3–4)	0.439
Biased learner (Utah *n* = 29, UTSW *n* = 71)			
Have attending address learner	4 (2–4)	3 (2–4)	0.121
Address it yourself	1 (1–1)	1 (1–3)	0.041
Ignore it	5 (5–5)	5 (5–5)	0.418
Collaborate with attending	3 (2–3)	2 (2–3)	0.011
Attending says to you only	3 (3–4)	4 (3–4)	0.065

*Note:* Lower median rank indicates stronger preference. Staff scenario uses available cases (*N* = 99 overall).

## 5 Discussion

This study examined how medical learners from different training stages, genders, and institutional contexts prefer to respond to biased statements in clinical learning environments. The results revealed significant institutional differences between participants from the University of Utah and UT Southwestern, particularly in how they preferred to address bias from patients, staff, peers, and other learners.

Participants from the University of Utah more frequently reported approaches involving direct engagement, such as addressing the bias themselves or involving the attending physician, whereas participants from UT Southwestern more commonly described approaches that were less confrontational or more passive. These findings suggest that institutional culture and training environments may shape learners' comfort and perceived responsibility in responding to biased behavior. Previous studies have emphasized the influence of institutional norms and perceived support on bias response behaviors [3, 5]. The current findings appear to echo this, highlighting the importance of institutional climate in shaping learners' actions.

In addition to institutional differences, gender‐based differences also emerged in the biased staff scenario. While no significant differences were found between male and female participants in how they preferred to respond to bias from patients, peers, or learners (all *p* > 0.05), men showed a significantly stronger preference for addressing the biased staff statement themselves (male median 1 [IQR 1–3] vs. female median 3 [IQR 1–4], *p* = 0.005). Conversely, women showed a significantly stronger preference for collaborating with an attending (female median 2 [IQR 1–2.2] vs. male median 2 [IQR 2–3], *p* = 0.034). This may reflect broader gendered expectations around assertiveness and advocacy in clinical settings, or differing perceptions of psychological safety [6]. These findings may not align with some previous literature suggesting that women in academic medicine often feel a stronger impetus to speak up against bias, possibly due to more frequent personal encounters with discrimination [4]. In contrast, men may be more inclined to defer or share responsibility when navigating such interactions.

Of note, elements such as cultural differences, prior bias training, and the influence of political pressures unique to each site were not studied, although there is no question any of these factors may have affected the responses.

These findings have several implications for medical education. First, they underscore the importance of tailoring bias response training not only to institutional culture but also to learner identity. Creating psychologically safe environments where all learners, regardless of gender, feel confident and supported in addressing bias is crucial. Second, institutions should consider incorporating scenario‐based discussions that address common dilemmas, such as how to handle bias from staff members, where power dynamics may complicate learners' decision‐making. Emphasizing a spectrum of response strategies—including personal action, collaboration, and escalation—may better equip learners with flexible, context‐sensitive tools.

While the study did not identify significant differences by training level in this analysis, the observed institutional and gender‐based trends emphasize the nuanced ways in which learners engage with bias. Further exploration of intersecting identities, such as race, ethnicity, or LGBTQ+ status, could reveal additional variation in preferred response strategies and inform more inclusive training approaches. Similarly, this study did not explore institutional culture or cultural mores specific to each respondent; this would be another area for future study.

## 6 Limitations

This study has some limitations. First, self‐reported data may introduce response bias. Second, survey design bias could have occurred if some questions were unclear, potentially skewing responses. Third, as a cross‐sectional study, it cannot establish causal relationships between the variables of interest. However, this study was descriptive and exploratory, rather than explanatory. Although the lack of specificity in the scenarios was intentional to allow the respondents to interpret bias as it applied to them, the use of just the term “bias” meant little can be drawn from a specific racist, sexist, or gender‐based bias. Our analysis did not examine the difference of institutional gender elements or size of respondent population, which may also have had an impact. We also did not stratify by training year (PGY); acknowledging that senior residents often prefer self‐management, this factor introduces variability that merits further evaluation. Finally, the limited sample size may reduce the generalizability of the findings to other settings or populations. Nonetheless, the goal of this study was to gain an in‐depth understanding of how bias is addressed, not to generalize the findings.

## 7 Conclusion

The study found that emergency medicine residents at two distinct institutions generally preferred active engagement in addressing biased statements, either by responding to themselves or involving the attending physician, especially when the bias came from colleagues or learners. Gender differences were observed for staff‐related bias: men more strongly preferred addressing the issue themselves, while women more strongly preferred collaborating with an attending. Institutional differences suggested that UT Southwestern residents more often favored attending‐led or collaborative approaches, whereas University of Utah residents more often favored addressing bias directly themselves. These findings support tailoring bias‐response training to both learner and institutional context.

## Author Contributions

**Jeff Druck:** conceptualization, methodology, data curation, investigation, validation, formal analysis, supervision, project administration, resources, writing – original draft, writing – review and editing, software. **Emad Awad:** data curation, formal analysis, writing – original draft, writing – review and editing. **William Harousseau:** conceptualization, methodology, project administration, writing – original draft, writing – review and editing. **Dustin Blake Williams:** conceptualization, methodology, investigation, supervision, project administration, writing – original draft, writing – review and editing.

## Conflicts of Interest

The authors declare no conflicts of interest.

## Supporting information


**Data S1:** Bias study.

## Data Availability

The data that support the findings of this study are available from the corresponding author upon reasonable request.
