# Supplementary material for: Gender and Institutional Differences in Preferred Resident Responses to Biased Statements in Medical Education: A Descriptive, Cross‐Sectional Analysis
Source: AEM Educ Train. 2026 Jun 9;10(3):e70206. doi: 10.1002/aet2.70206 (PMC13248764; doi:10.1002/aet2.70206)
Supplement: Supplementary file 1 — Data S1: Bias study. [file AET2-10-e70206-s001.pdf]

Default Question Block

Thank you so much for agreeing to take this survey; we estimate that it will take you less than five minutes to complete. The purpose of the study is to see how learners would like biased behavior addressed, based on the perpetrator. Although there are always modifiers (How well do I know this person? How significant is the biased behavior? etc), we ask you to use your best judgement and assume these scenarios to the best of your ability.

A patient makes a biased statement towards you, observed by a faculty member. How would you best like to address this issue? Rank in order with 1 being your most desired.

Have the attending collaborate with you about how to address it

Have the attending say something to you, but not the patient

Have the attending address it with the patient

Address it yourself

Ignore it

A staff member makes a biased statement towards you, observed by a faculty member. How would you best like to address this issue? Rank in order with 1 being your most desired.

Address it yourself

Have the attending address it with the patient

Have the attending collaborate with you about how to address it

Ignore it

Have the attending say something to you, but not the patient

A peer (same level) makes a biased statement towards you, observed by a faculty member. How would you best like to address this issue? Rank in order with 1 being your most desired.

Ignore it

Have the attending say something to you, but not the patient

Have the attending address it with the patient

Have the attending collaborate with you about how to address it

Address it yourself

Another learner, a year lower than you, makes a biased statement towards you, observed by a faculty member. How would you best like to address this issue? Rank in order with 1 being your most desired.

Have the attending address it with the patient

Have the attending collaborate with you about how to address it

Address it yourself

Have the attending say something to you, but not the patient

Ignore it

What best describes you?

- ◯ Medical Student
- ◯ Resident

What year are you?

- ◯ MS1
- ◯ MS2
- ◯ MS3
- ◯ MS4

What Department are you in?

- ◯ Anesthesiology
- ◯ Emergency Medicine
- ◯ Dermatology
- ◯ Family Medicine
- ◯ Internal Medicine
- ◯ Obstetrics/Gynecology
- ◯ Ophthalmology
- ◯ Otolaryngology
- ◯ Pathology
- ◯ Pediatrics
- ◯ Physical Medicine and Rehabilitation

- ○ Surgery
- ○ Urology
- ○ [ ] Other

What is the name of your institution

- ○ UT Southwestern
- ○ Harvard Medical School/ BI Deaconess
- ○ University of Utah
- ○ University of Wisconsin
- ○ Emory University
- ○ Mayo Clinic Florida
- ○ University of Colorado/ Denver Health
- ○ Eastern Carolina University
- ○ Thomas Jefferson University
- ○ [ ] Other

Have you seen bias from any of the following groups? Please select all that you have witnessed.

- ☐ [ ] Others
- ☐ Students
- ☐ Patient family members

- [ ] Residents
- [ ] Staff Members
- [ ] Patients
- [ ] Faculty

Choose one or more of the races you identify with- please pick as many apply to you

- [ ] American Indian or Alaska Native
- [ ] Asian
- [ ] Black or African American
- [ ] Hispanic or Latino
- [ ] Middle Eastern or North African
- [ ] Native Hawaiian or Pacific Islander
- [ ] White
- [ ] ____________ Other
- [ ] Prefer not to say

What sex were you assigned at birth?

- ○ Male
- ○ Female
- ○ ____________ Other

- ○ Prefer not to say

How do you describe yourself?

- ○ Male
- ○ Female
- ○ Non-binary / third gender
- ○ [ ] Prefer to self-describe
- ○ Prefer not to say

What is your sexual orientation?

- ○ Asexual
- ○ Bisexual
- ○ Heterosexual or straight
- ○ Gay/Lesbian
- ○ Pansexual
- ○ Queer
- ○ [ ] Other
- ○ Prefer not to say

Thank you for your participation.   Please let us know if you have other thoughts or concerns.

Powered by Qualtrics
